# Bone Mineral Density and Vascular Calcification in Children and Young Adults With CKD 4 to 5 or on Dialysis

**DOI:** 10.1016/j.ekir.2022.10.023

**Published:** 2022-11-02

**Authors:** Alexander D. Lalayiannis, Nicola J. Crabtree, Charles J. Ferro, David C. Wheeler, Neill D. Duncan, Colette Smith, Joyce Popoola, Askiti Varvara, Andromachi Mitsioni, Amrit Kaur, Manish D. Sinha, Lorenzo Biassoni, Simon P. McGuirk, Kristian H. Mortensen, David V. Milford, Jin Long, Mary D. Leonard, Mary Fewtrell, Rukshana Shroff

**Affiliations:** 1 Pediatric Nephrology, Birmingham Women’s and Children’s Hospitals, National Health Service Foundation Trust, Birmingham, UK; University College London Great Ormond Street Hospital Institute of Child Health, London, UK; 2 Nephrology, Birmingham Children’s Hospital, Birmingham, UK; 3 Densitometry Department, Birmingham Women’s and Children’s Hospitals National Health Service, Foundation Trust, Birmingham, UK; 4 Renal Unit, University Hospitals Birmingham, Birmingham, UK; 5 Department of Renal Medicine, University College London, London, UK; 6 Imperial College Healthcare National Health Service Trust, Renal and Transplant Center, London, UK; 7 Institute of Global Helath, University College London, London, UK; 8 St. George’s University Hospital National Health Service Foundation Trust, London, UK; 9 Department of Pediatric Nephrology, “P & A Kyriakou” Children’s Hospital, Athens, Greece; 10 Pediatric Nephrology, Manchester University National Health Service Foundation Trust, Manchester, UK; 11 Pediatric Nephrology, Evelina Children’s Hospital, London, UK; 12 University College London Great Ormond Street Hospital Institute of Child Health, London, UK; 13 Radiology Department, Birmingham Women’s and Children’s Hospitals National Health Service Foundation Trust, Birmingham, UK; 14 Department of Cardiac Imaging, University College London Great Ormond Street Hospital Institute of Child Health, London, UK; 15 Stanford University School of Medicine, Palo Alto, California, USA; 16 Pediatrics, Stanford University School of Medicine, Palo Alto, California, USA; 17 University College London Great Ormond Street Institute of Child Health, Population Policy and Practice, Childhood Nutrition Research Center, London, UK; 18 Great Ormond Street Hospital, London, UK

**Keywords:** chronic kidney disease, coronary artery calcification, carotid intima media thickness, peripheral quantitative CT scan, dialysis, dual-energy X-ray absorptiometry

## Abstract

**Introduction:**

Older adults with chronic kidney disease (CKD) can have low bone mineral density (BMD) with concurrent vascular calcification. Mineral accrual by the growing skeleton may protect young people with CKD from extraosseous calcification. Our hypothesis was that children and young adults with increasing BMD do not develop vascular calcification.

**Methods:**

This was a multicenter longitudinal study in children and young people (5–30 years) with CKD stages 4 to 5 or on dialysis. BMD was assessed by tibial peripheral quantitative computed tomography (pQCT) and lumbar spine dual-energy X-ray absorptiometry (DXA). The following cardiovascular imaging tests were undertaken: cardiac computed tomography for coronary artery calcification (CAC), ultrasound for carotid intima media thickness z-score (cIMTz), pulse wave velocity z-score (PWVz), and carotid distensibility for arterial stiffness. All measures are presented as age-adjusted and sex-adjusted z-scores.

**Results:**

One hundred participants (median age 13.82 years) were assessed at baseline and 57 followed up after a median of 1.45 years. Trabecular BMD z-score (TrabBMDz) decreased (*P* = 0.01), and there was a nonsignificant decrease in cortical BMD z-score (CortBMDz) (*P* = 0.09). Median cIMTz and PWVz showed nonsignificant increase (*P* = 0.23 and *P* = 0.19, respectively). The annualized increase in TrabBMDz (ΔTrabBMDz) was an independent predictor of cIMTz increase (*R*^2^ = 0.48, β = 0.40, *P* = 0.03). Young people who demonstrated statural growth (*n* = 33) had lower ΔTrabBMDz and also attenuated vascular changes compared with those with static growth (*n* = 24).

**Conclusion:**

This hypothesis-generating study suggests that children and young adults with CKD or on dialysis may develop vascular calcification even as their BMD increases. A presumed buffering capacity of the growing skeleton may offer some protection against extraosseous calcification.

## Graphical abstract

**Figure:**
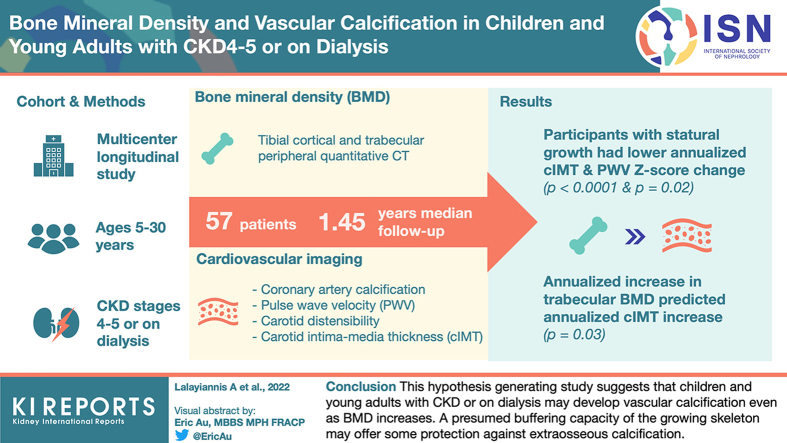

The growing skeleton is uniquely vulnerable to impaired mineralization in patients with CKD, manifesting as bone pain, deformities, and a 3-fold higher risk of fractures compared with healthy peers.^1^, ^2^, ^3^, ^4^ Just as healthy individuals require calcium (Ca) for skeletal mineralization,^5^ young patients with CKD, particularly growing children, who have higher serum Ca levels are shown to have a greater increase in bone mineral density (BMD).^6^ In contrast, patients with CKD, particularly those on dialysis, are at high risk of cardiovascular disease, which typically manifests as vascular calcification. High Ca intake and serum concentrations have been identified as key modifiable risk factors in the development and progression of vascular calcification.^7^

Decreasing BMD is linked with increasing vascular calcification in older adults with CKD.^8^, ^9^, ^10^ It is not known if bone demineralization is associated with vascular calcification in children and young adults with CKD. In older adults with CKD, bone demineralization may be considered an acceleration of physiological age-related osteoporosis, whereas in young people, the bone avidly accrues Ca as it grows and mineralizes. Our current practice is mostly based on extrapolations from adult studies, but this can be particularly harmful in children, leading to inappropriate treatment with potentially lifelong increase in fracture risk as well as cardiovascular disease. There are no longitudinal studies looking at BMD and vascular calcification simultaneously in children and young adults with CKD. Recognizing a gap in the literature, we performed a longitudinal follow-up study, performing a comprehensive assessment of bone and cardiovascular measures in a cohort of children and young adults with CKD stages 4 to 5 or on dialysis. Our hypothesis was that patients with increasing BMD z-scores do not have an increase in calcification scores.

The primary aim of the study was to examine the relationship between BMD and surrogate markers of vascular calcification in a young cohort with CKD. The secondary aim was to assess whether serial measurements of routinely used biomarkers such as Ca, phosphate, or parathyroid hormone are predictive of changes in BMD or vascular calcification.

## Methods

### Study Participants

We recruited young people (age 5–30 years) with CKD stages 4 to 5 (estimated glomerular filtration rate <30 ml/min per 1.73 m^2^) or on dialysis from 5 pediatric and 4 adult nephrology units. Given that bone mineral accrual continues until the third or fourth decade of life, when peak bone mass is reached^5^, young adults up to 30 years of age were included. We excluded patients with a functioning kidney transplant, patients with primary hyperoxaluria or cystinosis, patients with previous bisphosphonate treatment, or patients who would not have tolerated the scanning procedures. Informed written consent was obtained from all parents or caregivers and adult participants. Assent was obtained from children where appropriate. The study was approved by all local research ethics committees.

One hundred and thirty patients were identified and 112 agreed to participate. Twelve participants withdrew consent before taking part. A total of 100 children and young adults with CKD entered the study, and 57 were followed up after a median of 1.45 (1.25, 1.81) years. Cross-sectional data on bone health^11^ and subclinical cardiovascular disease^12^ in this cohort have been published (*N* = 100). All further analyses described pertain to the 57 participants who were followed up.

### Investigations Performed

Investigations were performed at the baseline study visit and at follow-up, with data presented as annualized changes in measures (Δ). BMD was assessed by tibial pQCT and lumbar spine DXA. Vascular measurements included cIMTz and distensibility, CAC by cardiac CT, carotid femoral pulse wave velocity, and pulse wave augmentation. A follow-up repeat cardiac CT scan was performed in all patients older than 18 years, but only in children younger than 18 years who had evidence of CAC on their baseline scan to minimize radiation exposure to children, according to the study protocol. Routine serum biomarkers were measured on nonfasting blood samples collected at the study visit or before a midweek hemodialysis session and analyzed in the patients’ respective hospitals. In addition, monthly serum biomarker measurements were performed as part of routine clinical care. These included serum ionized Ca, total Ca, phosphate, magnesium, bicarbonate, intact parathyroid hormone, 25-hydroxyvitamin D, and alkaline phosphatase (ALP). All imaging protocols have been published^11^^,^^12^ and are described in detail in the Supplementary Material together with details of the biomarker analysis.

Given the wide age range of participants, all age-related bone and cardiovascular measures are presented as z-scores and denoted by “z” after the respective measure. These z-scores reflect adjustments for age, sex, and/or height based on the changes with growth in the healthy pediatric population, allowing for comparison across all age groups. Lumbar spine DXA z-scores were expressed as bone mineral apparent density z-scores (BMAD).^13^ All lumbar spine DXA analyses are included in the Supplementary Material. Where normative data for young adults was not available (anthropometry, systolic and diastolic blood pressure [BP]), z-scores were calculated assuming a maximum age of 20 years. cIMTz and PWVz for young adults aged from 18 to 30 years were calculated using interpolation of the difference between 17 and 18 years in the reference data set,^14^^,^^15^ as published previously.^12^

### Statistics

All results are presented as a median with interquartile range or number and percentage. All biomarker concentrations were expressed as time-averaged levels over the course of the study. Because both the duration and extent of exposure above the upper limit of normal (ULN) for phosphate has been associated with higher morbidity and mortality,^16^ we expressed serum phosphate concentrations as an area under the curve, reflecting the time spent above ULN and the extent to which this threshold was exceeded (mmol × month/l).^16^ ULN was defined as 1.8 mmol/l (5.6 mg/dl) for 5- to 16-year-old patients and 1.45 mmol/l (4.5 mg/dl) for patients >16 years of age. Bone and cardiovascular measures were expressed as annualized changes as follows:

Δ change = (final visit value − baseline visit value)/follow-up time in years

Because tibial pQCT was the main bone imaging modality, growth was defined as tibial lengthening (>0 cm) between visits. Spearman rank testing was used for univariable correlations and Kruskal-Wallis analysis of variance test for non-normally distributed data with Dunn’s correction for multiple comparisons. Paired *t* testing was used for first and second visit comparisons. Paired Wilcoxon signed-rank testing was used to compare the CAC change in Agatston score because these data are nonparametric. Mann-Whitney *U* tests were used for between group nonparametric comparisons.

A series of multivariable linear regression models were built, with the dependent measure (ΔCortBMDz, ΔTrabBMDz, ΔcIMTz, Δdistensibility_z, ΔPWVz and Δaugmentation [Pulse wave augmentation annualized change]) as the dependent variables. All independent variables with univariable associations of *P* ≤ 0.15 were included in the multivariable models. Age and sex were not included as dependent variables because z-scores are adjusted for these. Being on dialysis or not was included as a binary dependent variable. Odds ratios are presented with 95% confidence intervals, and associated *P* values were calculated using the Fisher exact test. SPSS 27 (IBM) was used for all statistical analyses and Prism (GraphPad, San Diego, CA) to create figures. A two-sided *P* value of <0.05 was considered to indicate a statistically significant difference.

## Results

### Characteristics of the Participants

Demographics of the study population (that completed follow-up, *n* = 57) at baseline are shown in Table 1. The baseline characteristics of the whole cohort (*N* = 100) and their bone and vascular measures have been published^11^^,^^12^ and are summarized in Supplementary Tables S1 and S2. Fifty-seven patients were followed up after a median of 1.45 (1.25, 1.81) years. Forty-three patients were lost to follow-up because of transplantation (*n* = 26, 60.5%), restrictions to research activities during the COVID-19 pandemic (*n* = 16, 32.7%), or death (*n* = 1, 2.3%). Those lost to follow-up were older (15.84 vs. 12.71 years, *P* = 0.003; Supplementary Table S3), but otherwise comparable to the cohort studied. Four patients with CKD at baseline visit were started on kidney replacement therapy (1 on home hemodialysis and 3 on peritoneal dialysis) up to 2 months after their baseline visit.

**Table 1:** Patient characteristics at baseline (57 patients completed follow-up)

Participant characteristics at baseline	Values
Total, *N*	57
Age, yr	15.84 (12.56, 21.69)
20–30 yr	15 (26.32%)
5–19 yr	42 (73.68%)
Sex, female	23 (40.35%)
Race, Caucasian/Asian/Black/Other, *n*	27/17/12/1
Renal disease etiology, CAKUT/glomerular disease/cystic kidney diseases/vasculitides/other, *n*	28/9/6/4/10
Dialysis modality, CKD/HD/HDF/Home HD/PD, *n*	12/18/9/7/11
Years with eGFR <30 ml/min per 1.73 m^2^	7.01 (1.99, 10.28)
Dialysis vintage, yr	3.64 (0.58, 5.57)

CAKUT, congenital abnormalities of the kidneys and urinary tract; CKD, chronic kidney disease; eGFR, estimated glomerular filtration; HD, hemodialysis; HDF, hemodiafiltration; IQR, interquartile range; PD, peritoneal dialysis.

eGFR rate estimated by the Schwartz formula^17^ in children younger than 18 years.

Data are presented as median (IQR), *n* (%), or *n*.

### Longitudinal Changes in Bone Measures

Both TrabBMDz (−0.26 [−1.17, 1.93] to −0.38 [−1.47, 0.58], *P* = 0.01) and CortBMDz (−0.47 [−1.87, 0.16] to −1.13 [−2.76, −0.13], *P* = 0.26) decreased, but the reduction in CortBMDz was not statistically significant (Supplementary Table S4).

ΔTrabBMDz inversely correlated with both total Ca (*r* = −0.37, *P* = 0.006) and ionized Ca (*r* = −0.45, *P* = 0.01) on univariable associations. On multivariable regression, the only independent association with ΔTrabBMDz was TrabBMDz at baseline (*R*^2^ = 0.53, β = −0.70, *P* < 0.0001; Supplementary Table S6) (ΔBMAD regression in Supplementary Table S5) ΔCortBMDz correlated positively with both total Ca (*r* = 0.30, *P* = 0.03) and ionized Ca (*r* = 0.37, *P* = 0.04) on univariable associations. Ionized Ca and baseline CortBMDz were significant independent predictors of ΔCortBMDz (*R*^2^ = 0.23, β = 0.68, *P* = 0.01 and β = −0.55, *P* = 0.02, respectively; Supplementary Table S7).

### Longitudinal Changes in Vascular Measures

**Figure 1:**
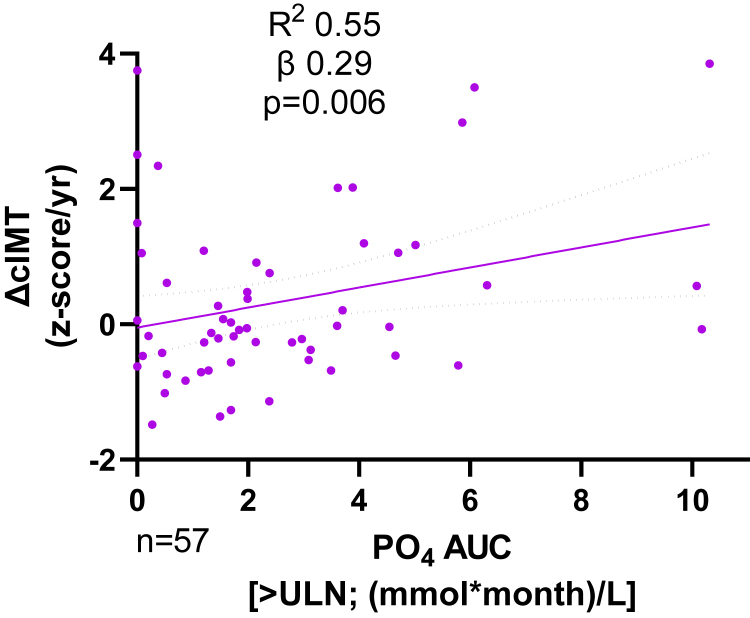
ΔcIMT correlation with phosphate AUC. *R*^2^, β, and *P* values from multivariable regression modeling (see Supplementary Table S8). ΔcIMT, annualized carotid intima media thickness z-score change; AUC, area under the curve; ULN, upper limit of normal.

Both the cIMTz and PWVz showed a nonsignificant increase over the study period (1.55 [0.93, 2.66] to 2.03 [1.23, 2.97], *P* = 0.10; and 1.08 [−0.42, 2.24] to 1.26 [0.25, 2.55], *P* = 0.11, respectively; Supplementary Table S8). ΔcIMTz was higher in dialysis patients compared with the CKD cohort (0.07 [−0.27, 1.15] vs. −0.56 [−0.78, −0.08], *P* = 0.004). Ionized Ca, total Ca, and ALP showed inverse univariable correlations with ΔcIMTz (*r* = −0.50, *P* = 0.004, *r* = −0.50, *P* < 0.0001; and *r* = −0.34, *P* = 0.01, respectively), whereas parathyroid hormone showed a positive univariable correlation (*r* = 0.36, *P* = 0.005) with ΔcIMTz. Patients with a positive ΔcIMTz change had higher parathyroid hormone values compared with those with a negative ΔcIMTz (6.7 × ULN vs. 2.8 × ULN or 37.5 vs. 15.7 pmol/l, *P* = 0.02). On multivariable linear regression, phosphate area under the curve >ULN and serum Ca were independent associations of ΔcIMTz (*R*^2^ = 0.55, β = 0.29, *P* = 0.006 and *R*^2^ = 0.55, β = −0.45, *P* < 0.001, respectively; Figure 1, Supplementary Table S9).

**Figure 2:**
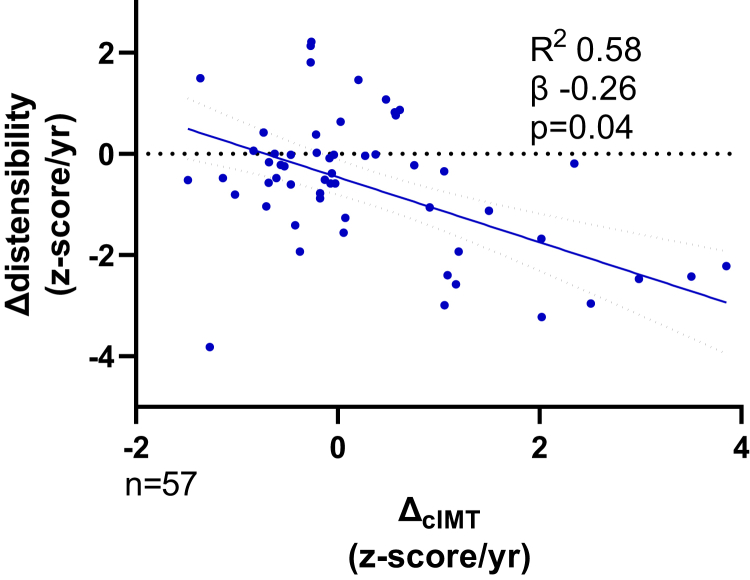
ΔcIMT correlation with Δdistensibility. R^2^, β, and *P* values from multivariable regression modeling. ΔcIMT, annualized carotid intima media thickness z-score change; Δdistensibility, annualized carotid distensibility z-score change.

Vascular stiffness in different arterial beds was assessed by a reduction in carotid artery distensibility or an increase in the carotid femoral PWV. There was a significant decrease in the distensibility_z (−1.22 [−2.17, −0.02] to −1.69 [−2.98, −0.70], *P* = 0.01) over the study period (Supplementary Table S8). On multivariable regression analysis, a decrease in Δdistensibility_z was predicted by increasing ΔcIMTz (*R*^2^ = 0.58, β = −0.26, *P* = 0.04, Figure 2), ALP (β = 0.26, *P* = 0.01), and ΔBMAD z-score (β = −0.25, *P* = 0.01) (Supplementary Table S11). The independent predictor of increase in arterial stiffness measured by PWV on multivariable regression was the annualized change in systolic BP (*R*^2^ = 0.45, β = 0.53, *P* = 0.001; Supplementary Table S10) and serum 25-hydroxyvitamin D (β = 0.28, *P* = 0.04). In addition, Δaugmentation correlated with Δsystolic BP (*r* = −0.35, *P* = 0.008) and Δdiastolic BP (*r* = −0.29, *P* = 0.03), but there were no independent associations on linear regression.

At the first study visit, 5 of 57 (9%) of the cohort who were followed up had CAC, and 10 of 18 (56%) who had a CT scan at the second study visit had CAC. The mean CAC score was 8.1 (range 0–412.6) at baseline and 42.61 (range 0–491.0) at follow-up. Of the patients with CAC at follow-up, 4 had CAC at baseline and 6 showed new-onset calcification (Supplementary Figure S1). The patients with an increase in CAC during follow-up had a higher phosphate area under the curve >ULN compared with those with no CAC (4.40 vs. 0.79 mmol × month/l, *P* = 0.04).

### Correlation Between Annualized BMD and Vascular Calcification Change

**Figure 3:**
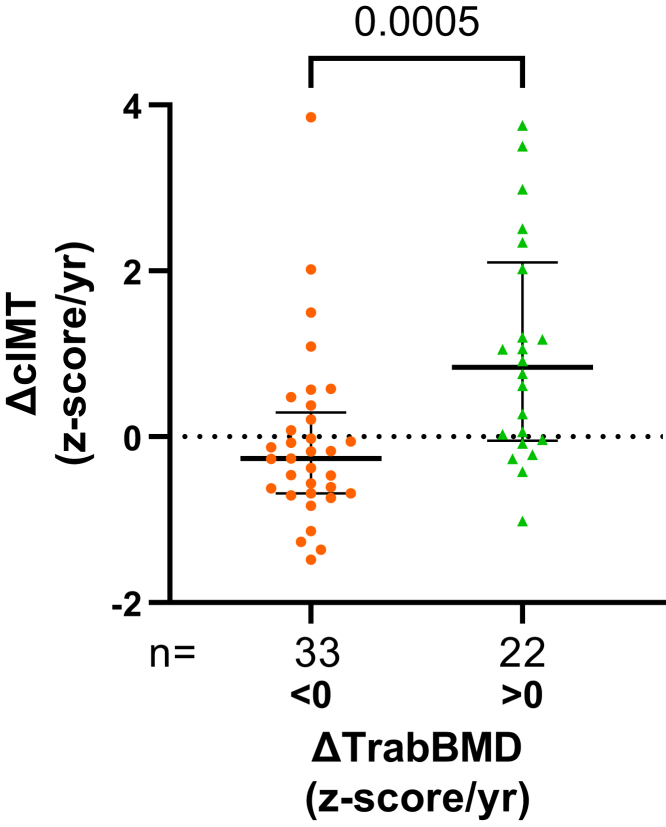
Median and interquartile range comparison of ΔcIMT between patients with increasing and decreasing ΔTrabBMD z-score. ΔcIMT, annualized carotid intima media thickness z-score change; ΔTrabBMD, annualized trabecular bone mineral density z-score change.

At follow-up, an increase in ΔTrabBMDz was an independent predictor of ΔcIMTz increase (*R*^2^ = 0.48, β = 0.40, *P* = 0.03; Supplementary Table S9, Model 1). The participants with a positive ΔTrabBMDz had a higher ΔcIMTz compared with those with no increase in ΔTrabBMDz (0.84 [−0.05, 2.10] vs. −0.26 [−0.68, 0.29], *P* = 0.005, Figure 3). Patients who had an increase in ΔTrabBMDz had 6-fold greater odds of having a concurrent increase in ΔcIMTz ([95% confidence interval 1.88–18.35], sensitivity 61.54% [42.53–77.57], specificity 79.31% [61.61–90.15], *P* = 0.003). Baseline cIMT regression model is presented in Supplementary Table S12.

### Effect of Linear Growth on Bone and Vascular Measures

Thirty-three patients (60%) demonstrated linear growth (>0 cm increase in tibial length; median growth 18.4 [15.9, 27.0] mm) during the study period. Annualized tibial length change correlated with annualized height change (*r* = 0.76, *P* < 0.0001) and higher ALP (*r* = 0.52, *P* < 0.0001). Patients with static growth were significantly older (25.5 [17.5, 28.0] vs. 13.2 [10.8, 15.5] years, *P* < 0.0001) and had lower serum ALP and Ca levels (112 [66, 139] vs. 234 [168, 291], *P* < 0.0001; and 2.40 [2.24, 2.49] vs. 2.44 [2.41, 2.58], *P* = 0.02, respectively), but there was no difference in the other biomarkers or medication intake between groups (Supplementary Table S13).

**Figure 4:**
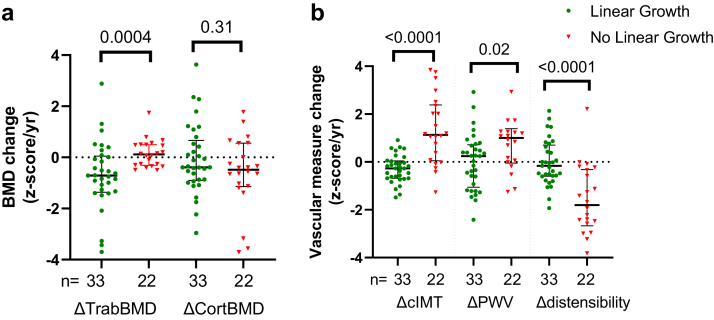
Comparison of (a) bone mineral density and (b) vascular measure changes (z-score/yr) for patients who demonstrated growth (tibial length increase) and no growth (static tibial length). ΔcIMT, annualized carotid intima media thickness z-score change; Δdistensibility, annualized carotid distensibility z-score change; ΔPWV, annualized pulse wave velocity z-score change; ΔTrabBMD, annualized trabecular bone mineral density z-score change.

ΔTrabBMDz was lower in patients with linear growth compared with participants with no growth (−0.71 vs. 0.11, *P* = 0.0004), implying undermineralized osteoid. ΔCortBMDz did not differ between groups (−0.39 vs. −0.48, *P* = 0.31; Figure 4a). Patients with linear growth had lower ΔcIMTz (−0.27 [−0.68, 0.04] vs. 1.13 [0.05, 2.39], *P* < 0.0001), lower ΔPWVz (0.24 [−1.06, 0.72] vs. 1.01 [−0.05, 1.39], *P* = 0.02), and higher Δdistensibility z-scores (−0.17 [−0.59, 0.70] vs. −1.81 [−2.67, −0.31], *P* < 0.0001) compared with participants with static growth (Figure 4b).

## Discussion

To our knowledge, this is the first prospective longitudinal study in a cohort of children and young adults with CKD stages 4 to 5 and on dialysis to concurrently examine changes in BMD alongside comprehensive measures of vascular calcification and stiffness. We have shown that even as BMD increases, vascular calcification can develop and progress in this young cohort with CKD: those with an increase in TrabBMDz were 6 times more likely to have an increase in cIMTz. Patients with linear growth showed an attenuated progression of all vascular measures, suggesting a buffering capacity of the growing skeleton. These hypothesis-generating data suggest that despite Ca accrual by the skeleton, excess Ca is deposited in soft tissues, leading to vascular calcification. A biomarker of bone mineralization that allows real-time evaluation of bone mineral balance, together with biomarkers of bone turnover, may determine the optimal Ca requirements of an individual, allowing normal bone mineralization without extraosseous calcification.

Osteoporosis and atherosclerosis were once thought to be unrelated diseases that are an inevitable part of the aging process. Recent studies suggest that bone demineralization and vascular calcification may be closely linked processes.^18^^,^^19^ Older adults with CKD demonstrate a similar but exaggerated “calcification paradox,”^20^ demonstrating a concurrent process of bone demineralization and ectopic soft-tissue calcification.^21^ In a longitudinal study measuring CAC and BMD in older dialysis patients, Malluche *et al.*^8^ showed that patients with the highest BMD loss had the greatest increase in CAC. Similar, albeit cross-sectional, studies have shown that both patients with CKD and those on dialysis who have a lower BMD have higher CAC scores^10^^,^^22^, ^23^, ^24^, ^25^, ^26^, ^27^ and a higher all-cause mortality.^22^ Importantly, the average age of patients in these studies was above 65 years, when age-related osteoporosis may have contributed to these processes.

Unlike the predominant bone resorption activity in older adults, the growing skeleton avidly accrues Ca; the Ca content of the skeleton increases from approximately 25 g at birth to approximately 1000 to 1200 g in adult males and females.^28^^,^^29^ Bone mineral accretion continues into the 30s, when peak bone mass (the amount of bone acquired by the end of skeletal development) is reached.^29^ Bone histomorphometry studies in children have demonstrated that defective mineralization was present as early as CKD stage 2, with the prevalence rate increasing to more than 90% in children on dialysis.^4^^,^^30^ In contrast, a mineralization defect was observed in only 3% of adults on dialysis.^31^ Therefore, CKD-MBD studies in older adults cannot be extrapolated to children and young people. There have been only 2 small cross-sectional studies in children with CKD to date. Preka *et al.*^32^ demonstrated that trabecular thickness by high-resolution pQCT was positively associated with diastolic and mean arterial BP. The second study, by Ziolkowska *et al.*,^33^ showed that cIMT correlated with lumbar spine BMD and total body DXA. Both studies were cross-sectional, making it impossible to determine the causal effect of BMD on vascular calcification, if any.

Our results suggest that linear growth may be a significant factor in the interplay between bone and blood vessels, and not merely ongoing mineralization. The young people with linear growth had lower ΔTrabBMDz compared with those with no linear growth. Mineralization of the osteoid scaffold lags behind formation of the bone matrix by osteoblasts by approximately 30 days.^34^ During periods of rapid growth in childhood and adolesence, mineralization follows the peak velocity of growth by 6 to 12 months.^35^ This leaves the bone compartment relatively undermineralized. In our cohort, the higher absolute ALP levels and negative ΔTrabBMDz demonstrated in growing children likely reflects this as-yet unmineralized osteoid. The process of ongoing bone mineral deposition may be a protective factor, buffering the vasculature from high-circulating Ca and phosphate. In adolescents, bone area increases rapidly around the time of and up to 5 years after peak height velocity and the bone mineral content continues to increase even afterward.^29^ Perhaps, once rapid linear growth ceases in late adolescence, the voracious absorption of minerals by the skeleton decreases, leading to increasing structural arterial wall changes. This may also be significant in the context of “catch up” growth that is achievable in children with CKD, with adequate nutrition and subsequent use of growth hormone treatment.^36^

Our data and studies in adult dialysis patients suggest that trabecular (rather than cortical) BMD loss is associated with vascular calcification.^8^^,^^22^^,^^37^ Trabecular bone is considered the more metabolically active bone compartment compared with cortical bone, accepting mineral ions from, and releasing them to, the circulation in response to hormonal stimuli.^35^ This is reflected in the mineralization process because new bone formation is completed in approximately 90 days in trabecular bone but requires approximately 120 days in cortical bone.^34^ Whereas cortical bone contains the majority of the bone mineral, the normal adult turnover rate is only 2% to 3% per year.^38^ Equally, newly formed bone with relatively low mineral content has a larger surface-to-volume ratio that allows exchange of Ca and phosphate with the extracellular fluid, highlighting the central role that the trabecular compartment plays in maintaining Ca and phosphate homeostasis.^38^ This may explain the inverse correlation between ionized Ca and Ca with ΔTrabBMDz, suggesting a greater exchange of Ca from the trabecular compartment to maintain normal serum levels.

With the exception of serum phosphate, the routinely used serum biomarkers did not correlate with BMD and vascular calcification changes. This is in keeping with earlier studies where we and others have shown that no biomarker individually or in combination is sufficiently robust to diagnose bone mineralization or turnover defects.^11^^,^^30^^,^^39^ Serum Ca levels are currently our only tool for estimating bone mineral balance. However, serum Ca accounts for <0.1% of total body Ca, and due to tight negative feedback control, does not reflect the total body Ca.^40^ Moreover, the mere presence of high serum Ca does not imply that it will be incorporated into bone, nor that it will be deposited in the vasculature. Instead, it is important to obtain a real-time estimation of bone mineral balance. We have shown that nonradioactive Ca isotopes that are naturally present in our food and water can be measured in serum, and their ratio (expressed as δ^44/42^Ca_serum_) is a significant and independent predictor of bone mineral balance in healthy children and young adults.^41^ This work has been extended to children with CKD and on dialysis in whom δ^44/42^Ca_serum_ values were the strongest predictor of total bone mineral content.^42^

Although, to our knowledge, this is the first study to follow changes in BMD and vascular measures concurrently in a young CKD and dialysis cohort, several limitations of our work must be acknowledged. As with all pediatric studies, surrogate measures of bone and cardiovascular outcomes were used. However, pQCT measurement of BMD has been associated with an increased fracture risk,^6^ and cIMT is a well-established surrogate for the extent of coronary artery disease, correlating with hard end points such as myocardial infarction and stroke in adults without CKD^43^ and cardiovascular events in patients with CKD^44^ and those undergoing dialysis.^45^ These intermediate end points must be interpreted with caution, and future studies must include key patient-level outcomes such as fractures that are commonly seen even in a young CKD cohort. In older adults, cIMT measurements may reflect both intimal thickening and atherosclerotic changes due to traditional Framingham risk factors. With the ultrasound imaging used, it is difficult to separate intimal and medial layers, but in children and young people, intimal thickening is rarely seen, and therefore, the cIMT increase can largely be attributed to medial layer changes.^46^^,^^47^ We could not perform bone biopsies, the current gold standard for determining mineralization changes in bone. Patient follow-up was affected by the COVID-19 pandemic when research studies were abruptly halted; this together with a high transplantation rate, contributed to significant loss to follow-up. Because those lost to follow-up were older, and the study protocol dictated that only children with CAC at baseline and all adults should have a cardiac CT at the second visit, the true incidence and progression of CAC may be underestimated or overestimated. In addition, all adults in the cohort were on hemodialysis, limiting our ability to examine bone and vessel changes in adults with CKD stages 4 to 5. Only 4 participants were on growth hormone treatment; therefore, we could not study its effects on bone mineralization or vascular calcification in this cohort. Finally, the follow-up was relatively short at a median of 1.5 years, and future longitudinal studies would benefit from a more prolonged follow-up period.

## Conclusion

In this hypothesis-generating study, we have shown that children and young adults with CKD or on dialysis develop vascular calcification even as BMD increases, with the most significant vascular changes in young people with no linear growth. One of the most challenging aspects of CKD-MBD management in young people is to reconcile the need for Ca and phosphate for optimal skeletal mineralization while avoiding an excess Ca intake that can lead to vascular calcification. An accurate estimation of the real-time changes in bone mineral balance may guide treatments based on the individual’s state of bone turnover and mineralization.

## Disclosure

All the authors declared no competing interests.

## Supplementary Material

Supplementary File (DOCX)

**Supplementary Methods.**

Investigations Performed.

**Figure S1.** Coronary artery calcification (Agatston score) at baseline and follow-up visits.

**Table S1.** Whole cohort patient characteristics at baseline (*n* = 100). Adapted from ^S5^ and ^S10^.

**Table S2.** Total cohort baseline bone and vascular measures. Adapted from ^S5^ and ^S10^.

**Table S3.** Demographics of patients who completed follow-up or were lost to follow-up***.***

**Table S4.** Bone measures at baseline, follow-up, between visit comparison and annualized difference.

**Table S5.** Annualized trabecular bone mineral density z-score change multivariable linear regression.

**Table S6.** Annualized Cortical bone mineral density z-score change multivariable linear regression modeling*.*

**Table S7.** Vascular measures at baseline, follow-up, between visit comparison and annualized difference*.*

**Table S8.** Annualized carotid intima media thickness z-score change multivariable linear regression model.

**Table S9.** Annualized pulse wave velocity z-score change multivariable linear regression model.

**Table S10.** Annualized carotid distensibility z-score change multivariable linear regression model.

**Table S11.** Medication intake and serum biomarker comparison for participants with tibial growth versus no growth.

**Table S12.** Multivariable linear regression with annualized bone mineral apparent density as the dependent variable.

**Table S13.** Multivariable linear regression model of baseline carotid intima media thickness z-score.
