# Supplementary material for: Bone Mineral Density and Vascular Calcification in Children and Young Adults With CKD 4 to 5 or on Dialysis
Source: Kidney Int Rep. 2022 Nov 2;8(2):265–73. doi: 10.1016/j.ekir.2022.10.023 (PMC9939315; doi:10.1016/j.ekir.2022.10.023)
Supplement: Supplementary File (DOCX) [file mmc1.docx]

Supplemental Material

Supplemental Table of Contents

[Investigations Performed 2](#_Toc120126925)

[Supplemental Table 1. Whole cohort patient characteristics at baseline (n=100) 4](#_Toc120126926)

[Supplemental Table 2. Total cohort baseline bone and vascular measures. Adapted from ^S5^ and  ^S10^ 6](#_Toc120126927)

[Supplemental Table 3. Demographics of patients who completed follow-up or were lost to follow-up. 7](#_Toc120126928)

[Supplemental Table 4. Bone measures at baseline, follow-up, between visit comparison and annualized difference. 8](#_Toc120126929)

[Supplemental Table 5. Multivariable linear regression with annualized Bone Mineral Apparent Density (BMAD) as the dependent variable 8](#_Toc120126931)

[Supplemental Table 6. Annualized trabecular bone mineral density z-score change multivariable linear regression 9](#_Toc120126932)

[Supplemental Table 7. Annualized Cortical bone mineral density z-score change multivariable linear regression modelling 11](#_Toc120126933)

[Supplemental Table 8. Vascular measures at baseline, follow-up, between visit comparison and annualized difference 12](#_Toc120126934)

[Supplemental Figure 1. Coronary artery calcification (CAC; Agatston score) at baseline and follow-up visits. 13](#_Toc120126935)

[Supplemental Table 9. Annualized carotid intima media thickness Z-score change (ΔcIMT) multivariable linear regression model. 14](#_Toc120126936)

[Supplemental Table 10. Annualized pulse wave velocity z-score change multivariable linear regression model 16](#_Toc120126937)

[Supplemental Table 11. Annualized carotid distensibility z-score change multivariable linear regression model 17](#_Toc120126938)

[Supplemental Table 12. Multivariable linear regression model of baseline carotid intima media thickness z-score.. 19](#_Toc120126939)

[Supplemental Table 13. Medication intake and serum biomarker comparison for participants with tibial growth vs no growth 20](#_Toc120126940)

# Investigations Performed

***Anthropometry***

Height was determined using a fixed wall stadiometer, and weight with a digital scale. Height, weight and body mass index (BMI) measurements were expressed as z-scores.

***Serum Biomarkers***

Routine serum biomarkers were measured on non-fasting blood samples collected at the study visit or prior to a mid-week hemodialysis session and analyzed in the patients’ respective hospitals. In addition, monthly serum biomarker measurements were performed as part of routine clinical care. These included serum ionized calcium (iCa), total calcium (Ca), phosphate (P), magnesium (Mg), bicarbonate, intact PTH (PTH), 25-hydroxyvitamin D [25OHD], and alkaline phosphatase (ALP). iCa was obtained by using Abbott iSTAT (USA) point of care analyzer with EG7+ cartridges. Due to different PTH assays used (Immulite [*Siemens Healthcare Diagnostics]* and Elecsys 2010 [*Roche Diagnostics*]), results have been expressed and analysed in multiples of the upper limits of normal (ULN), as well as in absolute values (pmol/L).

ALP values vary due to levels of bone activity and mineralization through life, and can be expressed as z-scores according to age and sex appropriate mean levels. We have used absolute ALP values primarily in the analysis, but have also expressed the ALP as z-scores for completion. The decision not to use ALP z-scores was because the CKD population may not have the same levels or timing of bone formation and mineralization and thus, using the absolute values reflects the level of bone activity, regardless of age or sex. For example, following a period of poor mineralization, there may be a catch up period on commencement of calcium based phosphate binders, that leads to exceptionally high z-scores for that patient’s age/sex. For this reason, we have kept the absolute ALP reporting in the main text, but have also analyzed with ALP as z-scores (ALPz) and included below in the Supplementary Materials. We have used the CALIPER trial to calculate z-scores, as per Uday et al ^S1, S2^.

Where absolute values of ALP have been significant on univariable correlation, ALP has been included in the regression model. Similarly, the regression model has then been repeated with ALPz if significance on univariable correlation, or without ALPz if no significance on univariable correlation.

***Bone and vascular imaging***

*Dual Energy X-ray Absorptiometry* All lumbar spine (LS) DXA scans were performed at the respective research centres according to the manufacturer’s protocol. The imaging was obtained according to the International Society for Clinical Densitometry (ISCD) guidelines using General Electric scanners (iDXA or Lunar) ^S3^. LS DXA z-scores were expressed as bone mineral apparent density (BMAD z-scores) ^S4^. As DXA can overestimate areal BMD in shorter or growth stunted people, it is necessary to adjust for height or bone size ^S3^. BMAD is a widely used method to adjust for size based on bone volume ^S4^. Young adults’ LS DXA z-scores were also adjusted for size and presented as BMAD z-scores as published previously ^S5^. Hip DXA was not performed in all participants as this imaging locus is not recommended for BMD estimation in growing children.

*Peripheral Quantitative Computed Tomography*  A scan of the non-dominant tibia was obtained by pQCT as per manufacturer’s instructions and ISCD guidelines ^S3, S6, S7^. The 3% metaphyseal and 38% diaphyseal sites were used for image acquisition of trabecular and cortical bone respectively, with results expressed as age-, sex-, race- and height adjusted z-scores derived from a reference dataset of 665 healthy children and young adults ages 5-35 years ^S6^.

*Carotid Intima Media Thickness (cIMT) and distensibility* cIMT measurements were obtained by ultrasound according to the Mannheim consensus ^S8^. The mean cIMT was calculated as the average IMT measurements of both carotids, 1-2 cm below the bifurcation using automatic software (Vivid iq, GE Healthcare, USA), and analyzed offline in a blinded fashion. M-mode was used for vessel systolic and diastolic diameter. cIMT measurements were expressed as Z-scores based on normative data from Doyon et al for children ^S9^ and the interpolation of the difference between 17 and 18 years for adults aged 18 to 30 years ^S10^.

*Cardiac Computed Tomography* Coronary artery calcification was assessed by CT (*Somatom Force;* *Siemens, Germany* or *GE Discovery 750HD, USA*) using the machines' standard Ca scoring protocol. Prospective ECG triggering was used to obtain images in diastole. Calcification was expressed as Agatston score ^S11^ and analyzed offline in a blinded fashion, confirmed independently by two observers (ADL and KHM) using Syngo Via software (*Siemens, UK*). It was decided *a priori* that a repeat cardiac CT scan would be performed in all patients above 18 years of age but only in children <18 years who had evidence of CAC on their baseline scan in order to minimize radiation exposure to children.

*Carotid-Femoral Pulse Wave Velocity (cfPWV) and Pulse Wave Augmentation* cfPWV and pulse wave augmentation index (AIx) were measured with the Vicorder Oscillometric PWV device (*SMART Medical*, *UK*) as previously described ^S10^.

All pQCT and DXA measures were performed by ADL or NJC, and all vascular measures by ADL.

# Supplemental Table 1. Whole cohort patient characteristics at baseline (n=100). Adapted from ^S5^ and ^S10^

|  | Total  (n = 100) | CKD  (n = 23) | Dialysis  (n = 77) | Between group comparison  (p-value) |
| --- | --- | --- | --- | --- |
| Age, years    5-18 years, n=(%)  19-30 years, n=(%) | 13.82 (10.68, 16.46)  79 (79)  21 (21) | 11.46 (6.80, 13.58)  23 (100)  0 | 14.25 (11.10, 21.95)  56 (73)  21 (27) | **0.002**    0.17  N/A |
| Sex, Female n=(%) | 44 | 6 (26.1) | 38 (49.4) | 0.06 |
| Race, n=  Caucasian/ Asian/ Black/ Other | 52/ 27/ 20/ 1 | 17/ 4/ 2/ 0 | 35/ 23/ 18/ 1 | **0.03** |
| Height Z-score | -1.09 (-1.93, -0.36) | -0.84 (-1.60, 0.04) | -1.42 (-2.02, -0.43) | 0.06 |
| Weight Z-score | -0.56 (-1.67, 0.20) | -0.21 (-1.02, 0.64) | -0.78 (-1.77, 0.02) | **0.02** |
| BMI Z-score | 0.14 (-0.88, 0.92) | 0.52 (-0.66, 1.28) | 0.01 (-0.95, 0.83) | 0.06 |
| Dialysis modality, n=  HD/HDF/home HD/PD | 44/ 14/ 3/ 16 | N/A | 44/ 14/ 3/ 16 | N/A |
| Phosphate binder therapy, n=  Calcium based/ Non-calcium based/ Both/ None  Calcium supplements (n=)  Total elemental Ca intake from medications (mg/kg/day)  Vitamin D analogs (n=)  alfacalcidol / paricalcitol/ calcitriol/ none) | 39/ 23/ 5/ 33  0  0.00 (0.00, 1.53)  69/6/1/24 | 16/ 1/ 0/ 6  0  1.09 (0.00, 2.09)  21/0/1/1 | 23/ 22/ 5 / 27  2  0.00 (0.00, 1.47)  48/6/0/23 | N/A  N/A  **0.03**  N/A |
| eGFR (ml/min/1.73m^2^) | N/A | 13.33 (9.72, 18.05) | N/A | N/A |
| Years with eGFR<30ml/min/1.73m^2^ | 5.58 (2.02, 10.10) | 3.68 (1.10, 8.81) | 5.63 (2.50, 10.45) | 0.09 |
| Dialysis vintage, years | 2.51 (0.75, 5.11) | N/A | 2.51 (0.75, 5.11) | N/A |

All data presented as median (IQR). N/A, not applicable; CAKUT, Congenital abnormalities of the kidneys and urinary tract; eGFR, estimated glomerular filtration rate estimated by Schwartz formula ^45^ in children under 18 years

# **Supplemental Table 2. Total cohort baseline bone and vascular measures.** Adapted from ^S5^ and ^S10^

|  | Total  (n=100) | CKD  (n=23) | Dialysis  (n=77) | Between group comparison  (p-value) |  |
| --- | --- | --- | --- | --- | --- |
| Bone measures (Z-score) | | | | | |
| Lumbar Spine BMADz | 0.37 (-0.83 to 1.04) | 0.71 ( -0.11 to 1.48) | 0.20 (-1.21 to 0.93) | **0.01** |  |
| Trabecular BMDz | 0.57 (-1.01 to 1.91) | 1.82 (-0.12 to 2.43) | -0.23 (-1.16 to 1.67) | **0.02** |  |
| Cortical BMDz | -0.35 (-1.57 to 0.29) | -0.15 (-0.47 to 0.31) | -0.72 (-1.9 to 0.28) | **0.04** |  |
| Vascular measures (Z-score) | | | | | |
| SBPz | 0.89 (0.03 to 1.67) | 0.40 (-0.10 to 1.13) | 0.96 (0.12 to 1.83) | **0.02** |  |
| DBPz | 0.72 (-0.14 to 1.36) | 0.50 (-0.18 to 1.11) | 0.87 (-0.04 to 1.45) | 0.30 |  |
| cIMTz | 2.17 (1.14 to 2.86) | 2.46 (1.04 to 2.76) | 2.01 (1.14 to 2.94) | 0.72 |  |
| PWVz | 1.45 (-0.16 to 2.57) | 0.61 (-0.78 to 2.23) | 1.52 (0.28 to 2.81) | **0.03** |  |
| Distensibilityz | -1.11 (-2.17 to -0.15) | -0.39 (-1.34 to 0.47) | -1.46 (-2.29 to -0.30) | **0.009** |  |
| CAC (Agatston Score) | range 0 to 412.6  10% had CAC | range 0 to 6.4  mean 0.28 ± SD 1.3  n=1 | range 0 to 412.6  mean 11.03± SD 63.4  n=9 | 0.36 |  |

BMAD; Bone mineral apparent density, BMD; bone mineral density, SBP; Systolic BP, DBP; Diastolic BP, cIMT; carotid intima media thickness, CAC; Coronary artery calcification. n=98 participants had Cardiac CT investigation at baseline.

Supplemental Table 3. Demographics of patients who completed follow-up or were lost to follow-up.

|  | **Total** | **Follow-up performed** | **Lost to follow-up** | **Between group difference** |
| --- | --- | --- | --- | --- |
| Total, n= (%) | n=100 | n=57 | n=43 | N/A |
| Age, years     5-19 years, n=(%)  20-30 years, n=(%) | 13.82 (10.68, 16.46)    80 (80)  20 (20) | 15.84 (12.56, 21.69)  42 (73.68)  15 (26.32) | 12.71 (8.75, 15.99)  37 (86.05)  6 (13.95) | **p=0.003** |
| Sex, Female n=(%) | 44 (44) | 23 (40.35) | 21 (48.84) | 0.42 |
| Race, n=  Caucasian/ Asian/ Black/ Other | 52/ 27/ 20/ 1 | 27/17/12/1 | 25/10/8/0 | 0.26 |
| Renal disease aetiology, n=  CAKUT/ Glomerular disease/ Cystic Kidney Diseases/ Vasculitides/ Other | 50/ 13/ 10/ 8/ 19 | 28/9/6/4/10 | 23/4/4/3/9 | 0.94 |
| Dialysis modality, n=  CKD/HD/HDF/Home HD/PD | 23/ 44/ 14/ 3/ 16 | 12/18/9/7/11 | 8/18/6/1/10 | 0.96 |
| Years with eGFR<30ml/min/1.73m^2^ | 5.58 (2.02, 10.10) | 7.01 (1.99, 10.28) | 4.80 (2.43, 10.00) | 0.63 |
| Dialysis vintage, years | 2.51 (0.75, 5.11) | 3.64 (0.58, 5.57) | 2.43 (0.95, 4.72) | 0.94 |

CAKUT; congenital abnormalities of the kidneys and urinary tract

**Bone Measures**

# Supplemental Table 4. Bone measures at baseline, follow-up, between visit comparison and annualized difference.

| Bone Imaging measure Z-scores | At Baseline  (n=55) | At Follow-up  (n=55) | Between visit difference  (p-value) | Annualized difference  (n=55) |
| --- | --- | --- | --- | --- |
| LS BMAD | -0.11 (-1.15, 0.76) | -0.60 (-2.04, 0.28) | **0.02** | -0.30 (-0.95, 0.35) |
| Trabecular BMD z-score | -0.26 (-1.17, 1.93) | -0.38 (-1.47, 0.58) | **0.01** | -0.30 (-0.84, 0.40) |
| Cortical BMD z-score | -0.47 (-1.87, 0.16) | -1.13 (-2.76, -0.13) | 0.26 | -0.39 (-0.99, 0.56) |

LS; Lumbar spine, BMAD; Bone mineral apparent density; BMD, Bone mineral density

# On DXA scan there was a significant decrease in LS BMADz [-0.11 (-1.15, 0.76) to -0.60 (-2.04, 0.28), p=0.02] from baseline to follow up (median, IQR).

On multivariable regression baseline BMADz (β -0.38, p=0.02) was an independent predictor of ΔBMADz (Supplemental Table 4).

Supplemental Table 5. Multivariable linear regression with annualized Bone Mineral Apparent Density (BMAD) as the dependent variable

| **Model Summary** | | | | | | | | |  |  |  |  |  |
| --- | --- | --- | --- | --- | --- | --- | --- | --- | --- | --- | --- | --- | --- |
| Model | | R | R Square | | Adjusted R Square | | Std. Error of the Estimate | |  |  |  |  |  |
| 1 | | 0.49^a^ | 0.24 | | 0.16 | | 0.81 | |  |  |  |  |  |
| a. Predictors: (Constant), PTH, Baseline BMAD, Mg, CKD/Dialysis | | | | | | | | |  |  |  |  |  |
| Model | | | | Unstandardized Coefficients | | | | Standardized Coefficients | | t | Sig. | 95.0% Confidence Interval for B | |
|  |  |  |  | B | | Std. Error | | Beta | |  |  | Lower Bound | Upper Bound |
| 1 | (Constant) | | | 2.06 | | 0.89 | |  | | 2.32 | 0.03 | 0.26 | 3.86 |
|  | CKD/Dialysis | | | -0.56 | | 0.34 | | -0.29 | | -1.65 | 0.11 | -1.25 | 0.13 |
|  | Baseline BMAD | | | -0.20 | | 0.08 | | -0.38 | | -2.43 | 0.02 | -0.37 | -0.03 |
|  | Mg | | | -1.35 | | 0.90 | | -0.23 | | -1.50 | 0.14 | -3.17 | 0.47 |
|  | PTH | | | 0.00 | | 0.01 | | -0.03 | | -0.18 | 0.86 | -0.01 | 0.01 |

BMAD; Bone mineral apparent density, Mg; magnesium

Supplemental Table 6. Annualized trabecular bone mineral density z-score change multivariable linear regression

| **Model Summary** | | | | | |  |  |  |  |  |  |
| --- | --- | --- | --- | --- | --- | --- | --- | --- | --- | --- | --- |
| Model | | R | R Square | Adjusted R Square | Std. Error of the Estimate |  |  |  |  |  |  |
| 1 | | 0.80^a^ | 0.63 | 0.53 | 0.69 |  |  |  |  |  |  |
| 2 | | 0.80^b^ | 0.63 | 0.53 | 0.68 |  |  |  |  |  |  |
| 1. Predictors: (Constant), Alkaline phosphatase, Magnesium, Baseline Trabecular BMD, CKD/Dialysis, annualized Cortical BMD change, ionized Calcium 2. As above, without Alkaline Phosphatase | | | | | |  |  |  |  |  |  |
| Model | | | Unstandardized Coefficients | | | | Standardized Coefficients | t | Sig. | 95.0% Confidence Interval for B | |
|  |  |  | B | | Std. Error | | Beta |  |  | Lower Bound | Upper Bound |
| 1. | (Constant) | | 5.28 | | 3.82 | |  | 1.39 | 0.18 | -2.63 | 13.20 |
|  | CKD/Dialysis | | 0.15 | | 0.31 | | 0.07 | 0.47 | 0.64 | -0.50 | 0.79 |
|  | Baseline Trabecular BMD | | -0.31 | | 0.06 | | -0.69 | -5.04 | <0.001 | -0.44 | -0.18 |
|  | Annualized cortical BMD change | | 0.29 | | 0.17 | | 0.28 | 1.72 | 0.10 | -0.06 | 0.64 |
|  | Ionized Calcium | | -4.87 | | 2.92 | | -0.31 | -1.67 | 0.11 | -10.92 | 1.17 |
|  | Magnesium | | 0.53 | | 1.33 | | 0.06 | 0.39 | 0.70 | -2.24 | 3.29 |
|  | Alkaline Phosphatase | | 0.00 | | 0.00 | | -0.02 | -0.12 | 0.91 | 0.00 | 0.00 |
| 2 | (Constant) | | 5.03 | | 3.09 | |  | 1.63 | 0.12 | -1.36 | 11.40 |
|  | CKD/Dialysis | | 0.16 | | 0.30 | | 0.77 | 0.452 | 0.61 | -0.460 | 0.77 |
|  | Baseline Trabecular BMD | | -0.31 | | 0.06 | | -0.69 | -5.16 | <0.001 | -0.44 | -0.19 |
|  | Annualized cortical BMD change | | 0.3 | | 0.14 | | 0.29 | 2.08 | 0.05 | 0.002 | 0.59 |
|  | ionized Calcium | | -4.67 | | 2.31 | | -0.29 | -2.02 | 0.06 | -9.44 | .11 |
|  | Magnesium | | 0.48 | | 1.26 | | 0.06 | 0.38 | 0.70 | -2.12 | 3.08 |

Supplemental Table 7. Annualized Cortical bone mineral density z-score change multivariable linear regression modelling

| **Model Summary** | | | | | | | |  |  |  |  |  |
| --- | --- | --- | --- | --- | --- | --- | --- | --- | --- | --- | --- | --- |
| Model | R | R Square | | | Adjusted R Square | Std. Error of the Estimate | |  |  |  |  |  |
| 1 | 0.67^a^ | 0.45 | | | 0.30 | 0.83 | |  |  |  |  |  |
| a. Predictors: (Constant), Magnesium, Baseline Cortical BMD, Annualized trabecular BMD change, CKD/Dialysis, ionized Calcium | | | | | | | |  |  |  |  |  |
| Model | | | Unstandardized Coefficients | | | | Standardized Coefficients | | t | Sig. | 95.0% Confidence Interval for B | |
|  |  |  | B | | | Std. Error | Beta | |  |  | Lower Bound | Upper Bound |
| 1 | (Constant) | | | -15.10 | | 4.37 |  | | -3.46 | 0.00 | -24.14 | -6.06 |
|  | CKD/Dialysis | | | -0.34 | | 0.41 | -0.18 | | -0.85 | 0.41 | -1.18 | 0.50 |
|  | Baseline Cortical BMD | | | -0.27 | | 0.11 | -0.55 | | -2.53 | 0.02 | -0.48 | -0.05 |
|  | Annualized trabecular BMD change | | | 0.33 | | 0.19 | 0.33 | | 1.74 | 0.10 | -0.06 | 0.71 |
|  | Ionized calcium | | | 10.51 | | 3.49 | 0.68 | | 3.01 | 0.01 | 3.29 | 17.72 |
|  | Magnesium | | | 2.21 | | 1.57 | 0.26 | | 1.41 | 0.17 | -1.03 | 5.45 |
| a. Dependent Variable: Annualized cortical Bone Mineral Density change | | | | | | | | | | | | |

**Vascular Measures**

Supplemental Table 8. Vascular measures at baseline, follow-up, between visit comparison and annualized difference

| Vascular measure Z-scores | At Baseline  n=57 | At Follow-up  n=57 | Between visit comparison (p-value) | Annualised difference |
| --- | --- | --- | --- | --- |
| cIMT | 1.55 (0.93, 2.66) | 2.03 (1.23, 2.97) | 0.10 | -0.06 (-0.50, 0.98) |
| PWV | 1.08 (-0.42, 2.24) | 1.26 (0.25, 2.55) | 0.11 | 0.39 (-0.55, 1.15) |
| Distensibility Coefficient | -1.22 (-2.17, -0.02) | -1.69 (-2.98, -0.70) | **0.01** | -0.48 (-1.48, 0.01) |
| CAC  (Agatston Score) | range 0 to 413  mean 8.10 ± SD 55.20  **n=57** | range 0 to 491  mean 42.61 ± SD 123.50  **n=18** | **0.002** | range 0 to 136.4  mean 12.68 ± SD 34.21  [4/10 had CAC previously  6/10 new CAC] |
| Augmentation  (%) | 6.83 (4.42, 10.00) | 6.42 (4.08, 10.50) | 0.89 | -0.80 (-3.18, 3.02) |
| Augmentation Index | 17.83 (11.42, 23.92) | 16.50 (12.08, 24.00) | 0.66 | -0.97 (-4.94, 6.79) |

cIMT; carotid intima media thickness, PWV; pulse wave velocity, CAC; coronary artery calcification

Supplemental Figure 1. Coronary artery calcification (CAC; Agatston score) at baseline and follow-up visits.

Agatston score in log-10 scale. P-value represents non-parametric paired t-testing.

| *Total cardiac CT scans, n= (%)* | *57 (100)* | *18 (100)* |
| --- | --- | --- |
| Total with CAC n= (%) | 5 (9) | 10 (56) |
| Children (5-18 yrs) with CAC | 1 (2) | 2 (11) |
| Children (5-18 yrs) without CAC | 41 (70) | 1 (6) |
| Young adults (19-30 yrs) with CAC | 4 (5) | 8 (44) |
| Young adults (19-30 yrs) without CAC | 11 (21) | 7 (39) |

Supplemental Table 9. Annualized carotid intima media thickness Z-score change (ΔcIMT) multivariable linear regression model. Models 1 and 2 include absolute Alkaline phosphatase as variables due to meeting the significance threshold on univariable correlation. The 1b and 2b models are the same models, without the Alkaline phosphatase as the Alkaline Phosphatase z-scores did not meet univariable significance.

|  | | | Unstandardized Coefficients | | Standardized Coefficients | t | Sig. | 95.0% Confidence Interval for B | |
| --- | --- | --- | --- | --- | --- | --- | --- | --- | --- |
|  |  |  | B | Std. Error | Beta |  |  | Lower Bound | Upper Bound |
| Model 1 | Adjusted R^2^ 0.48 | (Constant) | 5.12 | 5.11 |  | 1.00 | 0.33 | -5.45 | 15.70 |
|  |  | CKD/Dialysis | -0.21 | 0.35 | -0.10 | -0.61 | 0.55 | -0.92 | 0.50 |
|  |  | Baseline carotid intima media thickness z-score | -0.12 | 0.16 | -0.11 | -0.76 | 0.46 | -0.46 | 0.21 |
|  |  | Ionized calcium | -4.75 | 3.80 | -0.30 | -1.25 | 0.22 | -12.61 | 3.10 |
|  |  | Phosphate AUC >ULN | 0.28 | 0.08 | 0.47 | 3.31 | **0.003** | 0.11 | 0.46 |
|  |  | Alkaline phosphatase | 0.00 | 0.00 | -0.09 | -0.54 | 0.60 | 0.00 | 0.00 |
|  |  | PTH | 0.00 | 0.01 | 0.01 | 0.04 | 0.97 | -0.02 | 0.02 |
|  |  | Positive/Negative annualized trabecular bone mineral density change | 0.58 | 0.25 | 0.40 | 2.33 | **0.03** | 0.06 | 1.09 |
| Model 1b | Adjusted R^2^ 0.50 | (Constant) | 3.96 | 4.56 |  | 0.87 | 0.39 | -5.44 | 13.36 |
|  |  | CKD/Dialysis | -0.18 | 0.34 | -0.09 | -0.55 | 0.59 | -0.88 | 0.51 |
|  |  | Baseline carotid intima media thickness z-score | -0.13 | 0.16 | -0.11 | -0.78 | 0.44 | -0.46 | 0.21 |
|  |  | Ionized calcium | -3.96 | 3.44 | -0.25 | -1.15 | 0.26 | -11.05 | 3.14 |
|  |  | Phosphate AUC >ULN | 0.29 | 0.08 | 0.48 | 3.48 | **<0.001** | 0.12 | 0.46 |
|  |  | PTH | 0.00 | 0.01 | 0.01 | 0.05 | 0.96 | -0.02 | 0.02 |
|  |  | Positive/Negative annualized trabecular bone mineral density change | 0.57 | 0.24 | 0.39 | 2.32 | **0.03** | 0.06 | 1.07 |
| Model 2 | Adjusted R^2^ 0.55 |  | | | | | | | |
|  |  | (Constant) | 7.63 | 1.98 |  | 3.85 | 0.00 | 3.64 | 11.61 |
|  |  | CKD/Dialysis | 0.36 | 0.30 | 0.13 | 1.22 | 0.23 | -0.24 | 0.96 |
|  |  | Baseline carotid intima media thickness z-score | -0.16 | 0.13 | -0.13 | -1.21 | 0.23 | -0.42 | 0.11 |
|  |  | Total calcium | -3.40 | 0.75 | -0.45 | -4.55 | **<0.001** | -4.91 | -1.90 |
|  |  | Phosphate AUC >ULN | 0.14 | 0.05 | 0.29 | 2.85 | **0.006** | 0.04 | 0.25 |
|  |  | Alkaline phosphatase | 0.00 | 0.00 | -0.17 | -1.71 | 0.09 | 0.00 | 0.00 |
|  |  | PTH | 0.00 | 0.00 | 0.12 | 1.09 | 0.28 | 0.00 | 0.01 |
|  |  | Positive/Negative annualized trabecular bone mineral density change | 0.35 | 0.18 | 0.21 | 1.97 | **0.05** | -0.01 |  |
| Model 2b | Adjusted R^2^ 0.55 |  |  |  |  |  |  |  |  |
|  |  | (Constant) | 7.70 | 1.97 |  | 3.91 | 0.00 | 3.74 | 11.67 |
|  |  | CKD/Dialysis | 0.33 | 0.30 | 0.11 | 1.08 | 0.29 | -0.28 | 0.93 |
|  |  | Baseline carotid intima media thickness z-score | -0.24 | 0.13 | -0.19 | -1.93 | 0.06 | -0.49 | 0.01 |
|  |  | Total calcium | -3.46 | 0.75 | -0.47 | -4.64 | **<0.001** | -4.96 | -1.96 |
|  |  | Phosphate AUC >ULN | 0.16 | 0.05 | 0.32 | 3.26 | **<0.001** | 0.06 | 0.26 |
|  |  | PTH | 0.00 | 0.00 | 0.10 | 0.99 | 0.33 | 0.00 | 0.01 |
|  |  | Positive/Negative annualized trabecular bone mineral density change | 0.33 | 0.18 | 0.20 | 1.83 | 0.07 | -0.03 | 0.70 |

Supplemental Table 10. Annualized pulse wave velocity z-score change multivariable linear regression model

| **Model Summary** | | | | |  |  |  |  |  |
| --- | --- | --- | --- | --- | --- | --- | --- | --- | --- |
| Model | R | R Square | Adjusted R Square | Std. Error of the Estimate |  |  |  |  |  |
| 1 | 0.75^a^ | 0.56 | 0.45 | 0.83 |  |  |  |  |  |
| 2 | 0.74^b^ | 0.55 | 0.46 | 0.83 |  |  |  |  |  |
| 1. Predictors: (Constant), Vitamin D, Annualized SBP change, Annualized bone mineral apparent density z-score change, Annualized carotid Intima Media Thickness z-score change, Alkaline phosphatase, Magnesium, Baseline Pulse wave velocity z-score, CKD/Dialysis 2. As above, without Alkaline phosphatase | | | | |  |  |  |  |  |
| Model  1 |  | | Unstandardized Coefficients | | Standardized Coefficients | t | Sig. | 95.0% Confidence Interval for B | |
|  |  | | B | Std. Error | Beta |  |  | Lower Bound | Upper Bound |
|  | (Constant) | | 0.11 | 1.11 |  | 0.10 | 0.93 | -2.15 | 2.36 |
|  | CKD/Dialysis | | 0.42 | 0.36 | 0.18 | 1.17 | 0.25 | -0.31 | 1.15 |
|  | Baseline pulse wave velocity z-score | | -0.06 | 0.12 | -0.07 | -0.49 | 0.63 | -0.29 | 0.18 |
|  | Annualized SBP change | | 1.08 | 0.30 | 0.53 | 3.63 | **0.001** | 0.47 | 1.68 |
|  | Annualized carotid intima media thickness z-score change | | 0.17 | 0.18 | 0.13 | 0.97 | 0.34 | -0.19 | 0.54 |
|  | Annualized bone mineral apparent density z-score change | | 0.28 | 0.16 | 0.22 | 1.71 | 0.10 | -0.05 | 0.61 |
|  | Magnesium | | -1.00 | 1.09 | -0.13 | -0.92 | 0.36 | -3.22 | 1.21 |
|  | Alkaline phosphatase | | 0.00 | 0.00 | -0.11 | -0.85 | 0.40 | 0.00 | 0.00 |
|  | Vitamin D | | 0.01 | 0.00 | 0.28 | 2.15 | **0.04** | 0.00 | 0.01 |
| Model 2 | (Constant) | | 0.02 | 1.10 |  | 0.02 | 0.98 | -2.21 | 2.25 |
|  | CKD/Dialysis | | 0.40 | 0.35 | 0.17 | 1.12 | 0.27 | -0.32 | 1.12 |
|  | Baseline pulse wave velocity z-score | | -0.06 | 0.11 | -0.08 | -0.52 | 0.61 | -0.29 | 0.17 |
|  | Annualized SBP change | | 1.11 | 0.29 | 0.54 | 3.78 | **0.001** | 0.51 | 1.70 |
|  | Annualized carotid intima media thickness z-score change | | 0.16 | 0.18 | 0.11 | 0.89 | 0.38 | -0.20 | 0.52 |
|  | Annualized bone mineral apparent density z-score change | | 0.26 | 0.16 | 0.21 | 1.64 | 0.11 | -0.06 | 0.59 |

On multivariable regression analysis a decrease in Δdistensibility_z was predicted by increasing ΔcIMTz (R^2^ 0.58, β -0.26, p=0.04), ALP (β 0.26, p=0.01) and ΔBMADz (β -0.25, p=0.01) (Supplemental Table 10)

Supplemental Table 11. Annualized carotid distensibility z-score change multivariable linear regression model

| **Model Summary** | | | | | | | | |  |  |  |  |  |
| --- | --- | --- | --- | --- | --- | --- | --- | --- | --- | --- | --- | --- | --- |
| Model | | R | R Square | | Adjusted R Square | | Std. Error of the Estimate | |  |  |  |  |  |
| 1 | | 0.79^a^ | 0.63 | | 0.58 | | 0.97 | |  |  |  |  |  |
| 2 | | 0.77^b^ | 0.59 | | 0.55 | | 1.02 | |  |  |  |  |  |
| 1. Predictors: (Constant), Annualized bone mineral apparent density z-score change, Alkaline phosphatase, CKD/Dialysis, Total calcium, Baseline carotid distensibility z-score, Annualized carotid intima media thickness z-score change 2. As above, without Alkaline Phosphatase | | | | | | | | |  |  |  |  |  |
| Model | | | | Unstandardized Coefficients | | | | Standardized Coefficients | | t | Sig. | 95.0% Confidence Interval for B | |
|  |  |  |  | B | | Std. Error | | Beta | |  |  | Lower Bound | Upper Bound |
| 1 | (Constant) | | | -4.48 | | 2.64 | |  | | -1.70 | 0.10 | -9.78 | 0.83 |
|  | CKD/Dialysis | | | -0.68 | | 0.35 | | -0.20 | | -1.95 | 0.06 | -1.39 | 0.02 |
|  | Baseline carotid distensibility z-score | | | -0.41 | | 0.10 | | -0.39 | | -3.96 | **<0.001** | -0.62 | -0.20 |
|  | Total Calcium | | | 1.67 | | 1.04 | | 0.19 | | 1.60 | 0.12 | -0.43 | 3.77 |
|  | Alkaline phosphatase | | | 0.00 | | 0.00 | | 0.26 | | 2.63 | **0.01** | 0.00 | 0.00 |
|  | Annualized carotid intima media thickness z-score change | | | -0.32 | | 0.15 | | -0.26 | | -2.12 | **0.04** | -0.61 | -0.02 |
|  | Annualized bone mineral apparent density z-score change | | | -0.44 | | 0.17 | | -0.25 | | -2.56 | **0.01** | -0.79 | -0.09 |
| Model 2 | (Constant) | | | -2.57 | | 2.62 | |  | | -0.98 | 0.33 | -7.84 | 2.69 |
|  | CKD/Dialysis | | | -0.57 | | 0.36 | | -0.16 | | -1.57 | 0.12 | -1.31 | 0.16 |
|  | Baseline carotid distensibility z-score | | | -0.48 | | 0.10 | | -0.45 | | -4.58 | **<0.001** | -0.69 | -0.27 |
|  | Total Calcium | | | 1.00 | | 1.05 | | 0.11 | | 0.96 | 0.34 | -1.10 | 3.10 |
|  | Annualized carotid intima media thickness z-score change | | | -0.45 | | 0.15 | | -0.37 | | -3.06 | **0.004** | -0.74 | -0.15 |
|  | Annualized bone mineral apparent density z-score change | | | -0.40 | | 0.18 | | -0.23 | | -2.25 | **0.03** | -0.77 | -0.04 |

**Bone-Vascular Link**

At baseline for the whole n=100 cohort, cIMTz correlated with TrabBMDz (r=0.34, p<0.0001) and lumbar spine BMAD z-score (r=0.22, p=0.03).

On multivariable linear regression modelling for n=100, baseline TrabBMDz was an independent predictor of baseline cIMTz (R^2^ 0.10, β 0.34, p=0.001; Supplemental Table 11). LS BMAD showed a similar but weaker correlation with cIMTz when substituted in the same model (Supplemental Table 11, R^2^ 0.06, β 0.26, p=0.01). The odds of patients with a TrabBMD z-score >2 having a cIMT z-score >2 was 4.2 times higher [(95%CI 1.44 to 11.09), sensitivity 77.27% (56.56 to 89.88%), specificity 55.26% (44.10 to 65.92), p=0.008]. Cortical BMDz did not correlate with cIMTz. No correlations were seen with CAC.

Supplemental Table 12. Multivariable linear regression model of baseline carotid intima media thickness z-score. Models 1a and 2a include absolute Alkaline phosphatase as variables due to meeting the significance threshold on univariable correlation. The 1b and 2b models are the same models, without the Alkaline phosphatase as the Alkaline Phosphatase z-scores did not meet univariable significance.

| Model | | Unstandardized Coefficients | | Standardized Coefficients | t | Sig. | 95.0% Confidence Interval for B | |
| --- | --- | --- | --- | --- | --- | --- | --- | --- |
|  |  | B | Std. Error | Beta |  |  | Lower Bound | Upper Bound |
| Model 1a  Adjusted  R^2^ 0.10 | (Constant) | 1.15 | 1.60 |  | 0.72 | 0.47 | -2.02 | 4.32 |
|  | Baseline trabecular bone mineral density z-score | 0.17 | 0.05 | 0.34 | 3.39 | 0.001 | 0.07 | 0.27 |
|  | Total calcium | 0.31 | 0.64 | 0.05 | 0.49 | 0.63 | -0.96 | 1.58 |
|  | Alkaline phosphatase | 0.00 | 0.00 | -0.03 | -0.29 | 0.77 | -0.001 | 0.001 |
| Model 1b  Adjusted  R^2^ 0.11 | (Constant) | 1.13 | 1.56 |  | 0.73 | 0.47 | -1.96 | 4.22 |
|  | Baseline trabecular bone mineral density z-score | 0.17 | 0.05 | 0.35 | 3.51 | 0.001 | 0.08 | 0.27 |
|  | Total calcium | 0.30 | 0.63 | 0.05 | 0.48 | 0.63 | -0.95 | 1.55 |
| Model 2a  Adjusted  R^2^ 0.06 | (Constant) | -0.09 | 1.55 |  | -0.06 | 0.96 | -0.09 | 1.55 |
|  | Baseline Lumbar Spine BMAD z-score | 0.17 | 0.07 | 0.26 | 2.51 | 0.01 | 0.17 | 0.07 |
|  | Total calcium | 0.80 | 0.62 | 0.13 | 1.29 | 0.20 | 0.80 | 0.62 |
|  | Alkaline phosphatase | 0.00 | 0.00 | 0.07 | 0.73 | 0.47 | -0.001 | 0.001 |
| Model 2b  Adjusted  R^2^ 0.07 | (Constant) | 0.02 | 1.54 |  | 0.01 | 0.99 | -3.04 | 3.08 |
|  | Baseline Lumbar Spine BMAD z-score | 0.16 | 0.06 | 0.24 | 2.42 | 0.02 | 0.03 | 0.28 |
|  | Total calcium | 0.79 | 0.62 | 0.13 | 1.28 | 0.20 | -0.44 | 2.03 |

Supplemental Table 13. Medication intake and serum biomarker comparison for participants with tibial growth vs no growth

| Medication intake/ Serum Biomarker | Linear growth  (n=33) | Static growth  (n=22) | Between group difference  (p-values) |
| --- | --- | --- | --- |
| Elemental calcium intake from binders or supplements (mg/kg/day) | 11.79 (0.00, 30.29) | 4.23 (0.00, 17.84) | 0.36 |
| Alfacalcidol  (ng/kg/day) | 16.67 (2.67, 26.51) | 10.46 (4.53, 15.06) | 0.23 |
| Vitamin D (units/kg/day) | 0.00 (0.00, 37.34) | 0.00 (0.00, 36.39) | 0.92 |
| Calcium (mmol/L) | 2.44 (2.41, 2.58) | 2.40 (2.24, 2.49) | **0.02** |
| Magnesium (mmol/L) | 1.03 (0.91, 1.11) | 0.94 (0.89, 1.05) | 0.13 |
| Phosphate (mmol/L) | 1.55 (1.43, 1.75) | 1.58 (1.37, 1.84) | 0.95 |
| Phosphate AUC (mmol*month/L) | 1.69 (0.70, 3.32) | 2.07 (0.43, 4.25) | 0.61 |
| Bicarbonate (mmol/L) | 24.61 (21.71, 26.94) | 23.00 (21.84, 25.86) | 0.52 |
| Alkaline Phosphatase (units/L) | 233.9 (168.4, 291.3) | 112.2 (66.1, 139.3) | **<0.0001** |
| Alkaline Phosphatase  (z-score) | -0.12 (-1.03, 1.23) | 0.67 (-1.61, 2.91) | 0.70 |
| Intact parathyroid hormone (xULN) and (pmol/L) | x3.1 (x1.5, x6.6)  17.4 (8.4, 37.0) | x4.1 (x2.2, x12.9)  23.0 (12.3, 72.2) | 0.25 |
| Serum Vitamin D (units/L) | 96.50 (64.79, 127.80) | 92.53 (71.98, 107.10) | 0.57 |

Growth defined as >0cm lengthening of tibia during follow up period. ULN; Upper limit of normal range

S1. Uday S, Shaw NJ, Mughal MZ, et al. Monitoring response to conventional treatment in children with XLH: Value of ALP and Rickets Severity Score (RSS) in a real world setting. *Bone*. Oct 2021;151:116025. doi:10.1016/j.bone.2021.116025

S2. Colantonio DA, Kyriakopoulou L, Chan MK, et al. Closing the Gaps in Pediatric Laboratory Reference Intervals: A CALIPER Database of 40 Biochemical Markers in a Healthy and Multiethnic Population of Children. *Clinical Chemistry*. 2012;58(5):854-868. doi:10.1373/clinchem.2011.177741

S3. Crabtree NJ, Arabi A, Bachrach LK, et al. Dual-energy X-ray absorptiometry interpretation and reporting in children and adolescents: the revised 2013 ISCD Pediatric Official Positions. *J Clin Densitom*. Apr-Jun 2014;17(2):225-42. doi:10.1016/j.jocd.2014.01.003

S4. Crabtree NJ, Shaw NJ, Bishop NJ, et al. Amalgamated Reference Data for Size-Adjusted Bone Densitometry Measurements in 3598 Children and Young Adults-the ALPHABET Study. *J Bone Miner Res*. Jan 2017;32(1):172-180. doi:10.1002/jbmr.2935

S5. Lalayiannis AD, Crabtree NJ, Ferro CJ, et al. Routine serum biomarkers, but not dual-energy X-ray absorptiometry, correlate with cortical bone mineral density in children and young adults with chronic kidney disease. *Nephrology Dialysis Transplantation*. 2020;doi:10.1093/ndt/gfaa199

S6. Leonard MB, Elmi A, Mostoufi-Moab S, et al. Effects of Sex, Race, and Puberty on Cortical Bone and the Functional Muscle Bone Unit in Children, Adolescents, and Young Adults. *The Journal of Clinical Endocrinology and Metabolism*. 02/15 09/10/received 01/13/accepted 2010;95(4):1681-1689. doi:10.1210/jc.2009-1913

S7. Wetzsteon RJ, Kalkwarf HJ, Shults J, et al. Volumetric Bone Mineral Density and Bone Structure in Childhood Chronic Kidney Disease. *Journal of Bone and Mineral Research*. 2011;26(9):2235-2244. doi:10.1002/jbmr.427

S8. Touboul PJ, Hennerici MG, Meairs S, et al. Mannheim carotid intima-media thickness and plaque consensus (2004-2006-2011). An update on behalf of the advisory board of the 3rd, 4th and 5th watching the risk symposia, at the 13th, 15th and 20th European Stroke Conferences, Mannheim, Germany, 2004, Brussels, Belgium, 2006, and Hamburg, Germany, 2011. *Cerebrovasc Dis*. 2012;34(4):290-6. doi:10.1159/000343145

S9. Doyon A, Kracht D, Bayazit AK, et al. Carotid artery intima-media thickness and distensibility in children and adolescents: reference values and role of body dimensions. *Hypertension*. Sep 2013;62(3):550-6. doi:10.1161/hypertensionaha.113.01297

S10. Lalayiannis AD, Ferro CJ, Wheeler DC, et al. The burden of subclinical cardiovascular disease in children and young adults with CKD and on dialysis. *Clinical Kidney Journal*. 2021;doi:10.1093/ckj/sfab168

S11. Agatston AS, Janowitz WR, Hildner FJ, Zusmer NR, Viamonte M, Jr., Detrano R. Quantification of coronary artery calcium using ultrafast computed tomography. *J Am Coll Cardiol*. Mar 15 1990;15(4):827-32. doi:10.1016/0735-1097(90)90282-t
